# Enhanced cell survival and therapeutic benefits of IL-10-expressing multipotent mesenchymal stromal cells for muscular dystrophy

**DOI:** 10.1186/s13287-021-02168-1

**Published:** 2021-02-04

**Authors:** Yuko Nitahara-Kasahara, Mutsuki Kuraoka, Yuki Oda, Hiromi Hayashita-Kinoh, Shin’ichi Takeda, Takashi Okada

**Affiliations:** 1 Department of Biochemistry and Molecular Biology, Nippon Medical School, Bunkyo City, Tokyo Japan; 2 Division of Cell and Gene Therapy, Nippon Medical School, Bunkyo City, Tokyo Japan; 3 Department of Molecular Therapy, National Institute of Neuroscience, National Center of Neurology and Psychiatry, Kodaira, Tokyo Japan; 4 Laboratory of Experimental Animal Science, Nippon Veterinary and Life Science University, Musashino, Tokyo Japan; 5 Division of Molecular and Medical Genetics, Center for Gene and Cell Therapy, Institute of Medical Science, University of Tokyo, Minato-ku, Tokyo 108-8639 Japan

**Keywords:** Mesenchymal stromal cells, IL-10, DMD, Dental pulp stromal cells

## Abstract

**Background:**

Multipotent mesenchymal stromal cells (MSCs) are potentially therapeutic for muscle disease because they can accumulate at the sites of injury and act as immunosuppressants. MSCs are attractive candidates for cell-based strategies that target diseases with chronic inflammation, such as Duchenne muscular disease (DMD). We focused on the anti-inflammatory properties of IL-10 and hypothesized that IL-10 could increase the typically low survival of MSCs by exerting a paracrine effect after transplantation.

**Methods:**

We developed a continuous IL-10 expression system of MSCs using an adeno-associated virus (AAV) vector. To investigate the potential benefits of IL-10 expressing AAV vector-transduced MSCs (IL-10-MSCs), we examined the cell survival rates in the skeletal muscles after intramuscular injection into mice and dogs. Systemic treatment with IL-10-MSCs derived from dental pulp (DPSCs) was comprehensively analyzed using the canine X-linked muscular dystrophy model in Japan (CXMD_J_), which has a severe phenotype similar to that of DMD patients.

**Results:**

In vivo bioluminescence imaging analysis revealed higher retention of IL-10-MSCs injected into the hindlimb muscle of mice. In the muscles of dogs, myofiber-like tissue was formed after the stable engraftment of IL-10-MSCs. Repeated systemic administration of IL-10-DPSCs into the CXMD_J_ model resulted in long-term engraftment of cells and slightly increased the serum levels of IL-10. IL-10-hDPSCs showed significantly reduced expression of pro-inflammatory MCP-1 and upregulation of stromal-derived factor-1 (SDF-1). MRI and histopathology of the hDPSC-treated CXMD_J_ indicated the regulation of inflammation in the muscles, but not myogenic differentiation from treated cells. hDPSC-treated CXMD_J_ showed improved running capability and recovery in tetanic force with concomitant increase in physical activity. Serum creatine kinase levels, which increased immediately after exercise, were suppressed in IL-10-hDPSC-treated CXMD_J_.

**Conclusions:**

In case of local injection, IL-10-MSCs could maintain the long-term engraftment status and facilitate associated tissue repair. In case of repeated systemic administration, IL-10-MSCs facilitated the long-term retention of the cells in the skeletal muscle and also protected muscles from physical damage-induced injury, which improved muscle dysfunction in DMD. We can conclude that the local and systemic administration of IL-10-producing MSCs offers potential benefits for DMD therapy through the beneficial paracrine effects of IL-10 involving SDF-1.

**Supplementary Information:**

The online version contains supplementary material available at 10.1186/s13287-021-02168-1.

## Background

Multipotent mesenchymal stromal cells (MSCs) derived from the bone marrow are conventionally termed “adherent non-hematopoietic cells.” The cells express several cell-surface antigenic markers, including CD44, CD73, CD90, and CD105 [1]. MSCs can self-renew and differentiate into several different cell types. These include mesodermal cells, such as osteoblasts, chondrocytes, adipocytes, and myocytes [2–4] as well as non-mesodermal cells, such as hepatocytes [5], neural cells [6], and epithelial cells [7].

The multi-lineage potential of MSCs has been exploited for prospective use in therapies for various diseases. The cells can be easily expanded in culture and are non-tumorigenic. Furthermore, the use of MSCs as third-party materials in cell therapy reflects that MSCs are immune-privileged, unlike other stem cells or induced pluripotent stem cells (iPS), as they do not express human leukocyte antigen (HLA) class II, CD40, CD80, or CD86 molecules [8], and express only low levels of HLA class I. These cells are not lysed by natural killer cells or cytotoxic T lymphocytes [9]. MSCs can influence immune effector cell development, maturation, and function as well as reactive T cell responses through the production of bioactive cytokines and proteins [10, 11]. The mechanism underlying the immunosuppressive effects of MSCs is unclear. Nonetheless, their immunosuppressive properties have been exploited in clinical applications. MSCs are commercially authorized for the treatment of acute graft-versus-host disease (GVHD). MSCs are attractive candidates for cell-based strategies that target diseases with chronic inflammation, such as Duchenne muscular dystrophy (DMD) [11].

Interleukin-10 (IL-10) is an anti-inflammatory cytokine with anti-apoptotic properties [12] that modulates the inflammatory immune response. IL-10 reduces M1 macrophage activation and inhibits the production of pro-inflammatory cytokines such as interferon-gamma (IFN-γ), tumor necrosis factor-alpha (TNF-α), IL-1β, and IL-6 in inflamed tissues [13, 14]. IL-10 also reduces the expression of CD54, CD80, CD86, and major histocompatibility complex class II molecules, resulting in incomplete T cell signaling and induction of antigen-specific antibodies. We hypothesized that IL-10 could increase the typically low survival of MSCs by exerting a paracrine effect after transplantation. IL-10-expressing MSCs were previously developed using retroviral [15, 16] and lentiviral vectors [17, 18], and transcription activator-like effector nuclease (TALEN)-mediated gene editing [19]. They were observed to offer therapeutic benefits in collagen-induced inflammatory arthritis [20], and also prevented lung ischemia-reperfusion injury [15], endotoxin-induced acute injury [21], GVHD [16], traumatic brain injury [17], and left ventricular remodeling after myocardial infarction [19].

We developed a continuous IL-10 expression system of MSCs using an adeno-associated virus (AAV) vector. AAV vectors are safe and do not integrate into the host cell genome, and the risk of insertional mutagenesis is low. To investigate the potential benefits of using MSCs for treating muscle disease, we examined whether transduction with the IL-10-expressing AAV vector enhanced the survival rates of MSCs in skeletal muscles, and the potential advantages offered by MSC transplantation. Systemic treatment using MSCs derived from dental pulp (dental pulp stem cells, DPSCs) was comprehensively analyzed using the canine X-linked muscular dystrophy model in Japan (CXMD_J_), which has a severe phenotype similar to DMD in humans [22, 23]. DPSCs are similar to bone marrow MSCs, which show high expression of the surface markers CD29, CD73, CD90, and CD105, which are common stem cell markers in MSCs, but not CD34 or CD45. We have previously reported that DPSCs also express IL-10 and vascular endothelial growth factor (VEGF) at high levels and exhibit immunosuppressive activities [24, 25]. We evaluated whether DPSCs expressing IL-10 play an important role as a cell source for DMD therapy.

## Methods

### Animals

NOD/SCID mice were purchased from Nihon CLEA (Tokyo, Japan) and were housed at the National Center of Neurology and Psychiatry (Tokyo, Japan). All experiments using mice were performed in accordance with the guidelines approved by the Nippon Medical School and National Center of Neurology and Psychiatry (NCNP) Animal Ethics Committees. Beagle dogs and CXMD_J_ colony dogs were maintained at NCNP according to the NCNP standard protocol for animal care. Experiments were performed in accordance with the guidelines approved by the Ethics Committee for the Treatment of Laboratory Animals at NCNP.

### Cell preparation

MSCs derived from rat bone marrow were isolated and expanded as previously described [26]. For the experiments on dogs, healthy donor Beagle dogs were anesthetized using thiopental and isoflurane, and 1.0 mL of bone marrow fluid was collected. The CD271^+^ MSCs were enriched and cultivated using the MSC Research Tool Box-CD271 (LNGFR) containing CD271 (LNGFR)-PE and anti-PE microbeads for cell separation (Miltenyi Biotec GmbH, Bergisch Gladbach, Germany), as previously reported [27]. Human DPSCs were provided by JCR Pharmaceuticals (Hyogo, Japan). The cells were cultured in Dulbecco’s modified Eagle’s medium (DMEM; Thermo Fisher Scientific, Waltham, MA, USA) supplemented with 10% fetal bovine serum (FBS; Thermo Fisher Scientific) and 1% antibiotic-antimycotic solution (FUJIFILM Wako Pure Chemical Industries, Osaka, Japan) at 37 °C in a 5% CO_2_ atmosphere.

### Cell culture and gene transduction

To generate luciferase-expressing MSCs, MSCs isolated from Sprague-Dawley rat bone marrow [26] were transduced with vesicular stomatitis virus-glycoprotein (VSV-G)-pseudotyped retroviral vector encoding firefly luciferase [28]. Canine CD271^+^ MSCs were transduced with a luciferase-expressing retroviral vector, followed by transduction with enhanced green fluorescent protein (eGFP) or MyoD-expressing adenoviral vector (Ad C2-eGFP or Ad C2-MyoD), as reported previously [27]. To assess the long-term effects of IL-10 expression, MSCs or DPSCs were transduced with AAV1/eGFP or control AAV1/IL-10 vectors developed according to methods described previously [29, 30]. All cells were maintained in DMEM supplemented with 10% FBS, 100 U/mL penicillin, and 100 μg/mL streptomycin (Sigma-Aldrich, St. Louis, MO, USA). For transplantation, the cells were washed with PBS to completely remove the culture medium containing vectors.

### Transplantation of MSCs into mice

**Fig. 1:**
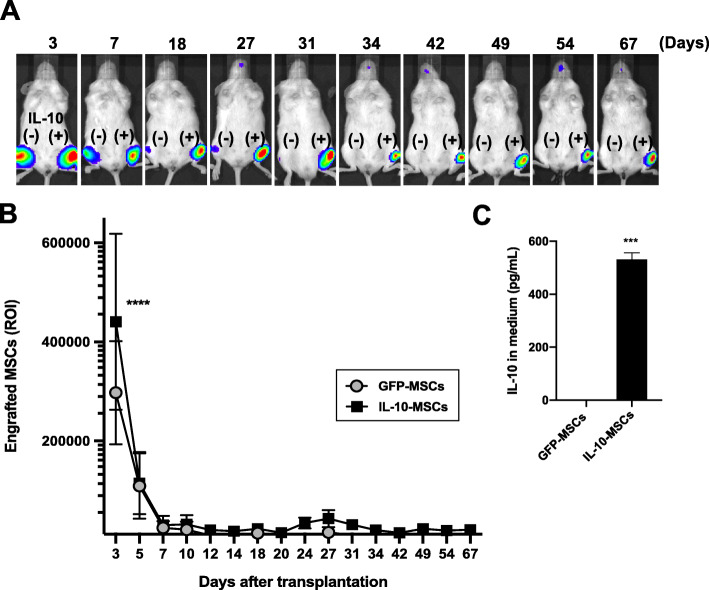
Extended engraftment of IL-10-MSCs. Luciferase-expressing rat MSCs were transduced with control AAV1/enhanced green fluorescence protein (GFP) or AAV1/IL-10 vector. **a** MSCs expressing GFP or IL-10 were injected into the right or left hindlimb muscle (IL-10 (−) or IL-10 (+)) of NOD/SCID mice. In vivo bioluminescence imaging of mice treated with MSCs expressing GFP or IL-10 revealed the appearance of luciferase signals between 3 and 67 days after intramuscular injection into the right or left hindlimb muscle: GFP-MSCs, IL-10 (−), or IL-10-MSCs, IL-10 (+). **b** Monitoring of the quantitative luciferase counts at the GFP- or IL-10-MSC-injected site by imaging analysis conducted between 3 and 67 days after treatment (*n* = 6–3). **c** Quantitative measurement of IL-10 levels in the MSC culture medium using ELISA 7 days after transduction with AAV1/GFP or AAV1/IL-10 (*n* = 3). Data are presented as mean ± SD, and statistical differences between GFP-MSCs and IL-10-MSCs are indicated, ^***^P < 0.001, ^****^P < 0.0001, *t*-test are indicated

Luciferase-expressing rat MSCs (Luc-MSCs, 5.0–10.0 × 10^6^ cells) were injected intramuscularly into the right or left hindlimb muscle of NOD/SCID mice pretreated with cardiotoxin (10 μM, Merck KGaA, Darmstadt, Germany) 1 day before the treatment. AAV1/IL-10- or eGFP-vector-transduced Luc-MSCs (1.0 × 10^7^ cells, Fig. 1) were intramuscularly injected into the right (eGFP-MSCs) and left (IL-10-MSCs) hindlimb muscles of NOD/SCID mice.

### In vivo imaging analysis

After the injection of luciferase-expressing rat MSCs on day 0 of the experiment, in vivo luminescence images were acquired periodically to assess the engraftment efficiency and cell survival in the transplanted mice. Prior to imaging, the mice were anesthetized by inhalation of 2.0% isoflurane and oxygen and injected intraperitoneally with 150 mg luciferin (Summit Pharmaceuticals International Corp., Tokyo, Japan.) per kilogram body weight. In vivo images were acquired using the IVIS charge-coupled-device camera system (Xenogen Corp., Alameda, CA, USA) at multiple time points (0, 3, 7, 18, 27, 31, 34, 42, 49, 54, and 67 days after transplantation). The region of interest (ROI) luminescence signals from individual MSC-injected sites were measured using the Living Image® 3.2 software package (Xenogen Corp.).

### Transplantation into dogs

AAV1/IL-10-transduced Luc-CD271^+^ MSCs derived from a healthy dog (2.4–2.7 × 10^7^ cells/2 mL) were injected into the muscles of healthy Beagle dogs. Five days before the treatment, muscle degeneration were induced in the tibialis anterior (TA) muscles by injecting 10 nmol/kg cardiotoxin (C9759, Sigma-Aldrich) under anesthesia. For analgesia treatment, 0.02 mg/kg of buprenorphine hydrochloride (Lepetan, Otsuka Pharmaceutical, Tokyo, Japan) was injected intramuscularly before the effect of general anesthesia wore off. On days 0 and 50 of the experiment, the MSCs were injected into pretreated muscles without using immunosuppressants. The injected muscles were then biopsied at 4 weeks after the treatment, or the animals were sacrificed at 8 weeks after transplantation. The dogs underwent periodic veterinary examinations during the experiments.

hDPSCs or AAV1/IL-10-transduced hDPSCs (4.0 × 10^6^ cells/mL/kg body weight at a rate of 1 mL/min) were administered via intravenous injection into CXMD_J_ that were pretreated with polaramine (chlorpheniramine maleate, 0.15 mg/kg) using nine injections at 2-week intervals (Table 1). After each injection, the activity, heart rate, respiratory rate, and signs of abnormalities were carefully monitored. Weight measurement and blood tests were performed weekly to examine the side effects of repeated cell treatment.

**Table 1:** Summary of transplantation experiments

Dog ID	Sex	Age^a^	BW^b^	Cell	Cell numbers	Interval	Injection numbers	Route
4502MN	M	51	11.3	IL-10-CD271^+^MSCs, MyoD-CD271^+^MSCs	2.5 × 10^7^ cells	–	1	i.m.
5601MN	M	40	11.2	IL-10-CD271^+^MSCs, MyoD-CD271^+^MSCs	4.0–10.0 × 10^6^ cells	–	2	i.m.
14103MN	M	3	5.0	–	–	–	–	–
14102MA	M	3	3.3	–	–	–	–	–
14105MA	M	3	3.4	hDPSCs	4.0 × 10^6^ cells/kg	2 weeks	9	i,v.
14108MA	M	3	3.5	IL-10-hDPSCs	4.0 × 10^6^ cells/kg	2 weeks	9	i,v.

*M* male, *i.m* intramuscular injection, *i.v*. intravenous injection

^a^Age at injection (months)

^b^*BW* body weight at first injection (kg)

For biopsy and necropsy, the individual muscles were sampled for tendon-to-tendon dissection, divided into several fragments, and immediately frozen in liquid nitrogen-cooled isopentane for histological analysis. Whole muscle tissue homogenates were prepared using a POLYTRON homogenizer (150–180 min^− 1^) and Multi-Beads Shocker (Yasui Kikai Corp. Osaka, Japan).

### Blood test

The dogs underwent periodic veterinary examinations at 1–2-week intervals until sampling. Hematological and serum biochemical testing for creatine kinase (CK) was performed using a model F-820 semi-automated hematology analyzer (Sysmex, Hyogo, Japan). The levels of serum alanine aminotransferase (ALT), aspartate aminotransferase (AST), and blood urea nitrogen (BUN) were determined using a DRI-CHEM3506 automated analyzer (Fuji Film, Tokyo, Japan).

### Histopathological and immunohistochemical analyses

Samples from MSC-treated TA muscles were collected and immediately frozen in liquid nitrogen-cooled isopentane. Five mice from each group were used for analysis at each time point. Transverse cryosections 8 μm in thickness prepared from the skeletal muscles were stained with H&E using standard procedures. For immunohistochemical analyses, thick cryosections were fixed in acetone for 5 min at − 20 °C. The tissue sections were then blocked using 0.5% bovine serum albumin (BSA) in PBS. The following antibodies were used for antigen detection at 1:40–1:50 dilutions: rabbit anti-firefly luciferase (ab21176; Abcam Plc., Cambridge, UK) and mouse anti-dystrophin (NCL-DYS3, Leica, Wetzlar, Germany). These antibodies were diluted using 0.5% BSA in PBS and used to treat the cells or tissue sections overnight at 4 °C. The tissue sections were washed with PBS and then probed with Alexa 568-conjugated anti-rabbit IgG antibodies (Thermo Fisher Scientific) and Alexa 488-conjugated anti-mouse IgG antibodies (Thermo Fisher Scientific) at 1:250–1:100 dilution for 1 h at 4 °C. The coverslip slides were washed with PBS and mounted in Vectashield (Vector Laboratories Inc., Burlingame, CA, USA) with 4,6-diamidino-2-phenylindole (DAPI). Immunofluorescence analysis was performed using an IX71 fluorescence microscope (Olympus, Tokyo, Japan).

To confirm the presence of transplanted cells at the injection sites, the MSCs were labeled with luciferase or GFP. The tissue sections were incubated in a solution of 3% H_2_O_2_ to block endogenous peroxidase. The nonspecific binding sites were blocked using a 2% BSA solution. The tissue sections were probed with primary antibodies for 1 h and then treated using the 3,3′-diaminobenzidine (DAB) substrate kit (Vector Laboratories Inc.) containing horseradish peroxidase (HRP) as an enzyme indicator. The slices were then stained with DAB chromogen to determine the form of the brown-antigen reaction product. The tissue sections were visualized using an IX71 microscope (Olympus).

### ELISA

The IL-10 expression levels were measured in the FBS-free MSC culture medium after 2 days of incubation, in the TA muscle lysate, and in the serum obtained from animals using the Quantikine ELISA mouse or canine IL-10 Immunoassay (Thermo Fisher Scientific) and canine IL-6 (R&D Systems Inc.) and the collagen type III Immunoassay (Cloud-clone Corp. TX, USA), according to the manufacturers’ recommendations. The final values were normalized to the protein concentrations, which were measured using the Pierce® BCA Protein Assay Kit (Thermo Fisher Scientific).

### Luciferase reporter assays

Luciferase reporter assays were performed to evaluate the retention of Luc-MSCs in the TA muscle. Firefly luciferase activity was tested in whole tissue homogenates using the Bright-Glo™ Luciferase Assay System (Promega Corporation, Madison, WI) according to the manufacturer’s instructions. Luciferase levels were measured using a Varioskan LUX Multimode Microplate Reader (Thermo Fisher Scientific). Protein concentrations were measured using a Pierce® BCA Protein Assay Kit (Thermo Scientific Pierce, Rockford, IL, USA). Three independent experiments were performed in duplicate.

### Biodistribution of MSCs

The tissue samples were disrupted in a Multi-Beads Shocker (Yasui Kikai Co., Ltd., Osaka, Japan). DNA was extracted from the tissue suspensions using a DNeasy Blood and Tissue kit (QIAGEN, Valencia, CA) and quantified using a NanoDrop spectrophotometer (Thermo Fisher Scientific). Real-time qPCR was performed using 125 ng of DNA in a total volume (20 μL) containing DNA Master SYBR Green I kit (Roche Diagnostics, Basel, Switzerland) and primers for *Alu* or murine glyceraldehyde 3-phosphate dehydrogenase (*Gapdh*). The primer sequences used were as follows: human *Alu*, 5′-GTCAGGAGATCGAGACCATCCC-3′ (forward) and 5′-TCCTGCCTCAGCCTCCCAAG-3′ (reverse); murine *Gapdh*, 5′-GATGACATCAAGAAGGTGGTGA-3′ (forward) and 5′-TGCTGTAGCCGTATTCATTGTC-3′ (reverse). The PCR conditions were as follows: 95 °C for 2 min, followed by 40 cycles at 95 °C for 15 s, 68 °C for 30 s, and 72 °C for 30 s. The standard was generated by adding 10-fold serial dilutions of human DPSCs to determine the number of human DPSCs in 125 ng of DNA that was used in the real-time PCR for each organ sample. We extrapolated the quantity of DNA isolated from each organ to determine the number of human DPSCs per organ.

### Proteome cytokine/cytokine array

The FBS-free DPSC culture medium was collected after 2 days of incubation for array analysis. The relative expression of cytokines and chemokines in the culture medium was quantified using the Proteome Profiler™ Array (Mouse Cytokine Array, Panel A; R&D Systems Inc.), as previously described [28]. To achieve maximum assay sensitivity, the blots were incubated overnight with plasma. Enhanced chemiluminescence incubation was performed for 5 min using the Super Signal West Femto Chemiluminescence Kit (Thermo Scientific Pierce), and the samples were imaged and analyzed using the Image Quant LAS 4000 coupled with Image Quant TL software (GE Healthcare Japan, Tokyo, Japan) and Image J software (NIH, Bethesda, MD).

### Locomotor activity analyses

The physical activity levels of CXMD_J_ and littermate normal dogs used as controls were monitored during the experimental period using an infrared sensor system (Supermex, Muromachi Kikai Co., Ltd., Tokyo, Japan), as previously described [31]. These systems monitor and enumerate all spontaneous movements. The average of all counts of spontaneous locomotor activity in animals determined over 5 days and nights (12 h light/dark cycles) was calculated. Furthermore, we measured the 15-m running time of normal and CXMD_J_ littermates during the experimental period. The running speed was averaged four times.

### Magnetic resonance imaging (MRI)

CXMD_J_ anesthetized by injection (20 mg/kg) were intubated using an endotracheal tube, and general anesthetization was maintained using an inhalational mixture of 2 to 3% isofluorane and oxygen. The heart rate and oxygen saturation levels were monitored continuously. Images of the T2-weighted and fat-saturated T2-weighted series were captured using a method described in a previous study [32]. We examined the crus muscles of the lower limbs using a superconducting 3.0-Tesla MRI device (MAGNETOM Trio; Siemens Medical Solutions, Erlanger, Germany) with an 18-cm diameter/18-cm length human extremity coil. The images were analyzed quantitatively using the Syngo MR2004A software (Siemens Medical Solutions), as previously reported [32, 33]. Briefly, the ROIs were selected to avoid flow artifacts and large vessels, and the signal intensities were measured for these ROIs. The SNRs for each ROI were calculated using the following equation: SNR = signal intensity/SDair, where SDair is the standard deviation (SD) of the background noise. The average SNR (Ave SNR) was calculated using the equation described in a previous report [33]. The right and left TA muscles, EDL, gastrocnemius medial head, GL, flexor digitorum superficialis, flexor digitorum longus, and flexor hallucis longus muscle were examined.

### Statistical analyses

Data are presented as the mean ± SD. Differences between two groups were assessed using unpaired two-tailed *t*-tests. Multiple comparisons between three or more groups were performed using ANOVA (*n* = 3–6). Statistical significance was defined as ^*^P < 0.05, ^**^P < 0.01, ^***^P < 0.001, and ^****^P < 0.0001 and was calculated using Microsoft Excel (Microsoft, Redmond, WA, USA) and GraphPad Prism 8 (GraphPad, La Jolla, CA, USA).

## Results

### Enhanced engraftment of IL-10-expressing MSCs

We investigated the effects of IL-10 after MSC injection into the skeletal muscle. In vivo bioluminescence imaging analysis showed that IL-10-expressing MSCs tended to exhibit higher retention in the hindlimb muscle of NOD/SCID mice than normal MSCs 4 days after injection; however, upon quantitative analysis, the difference was not found to be significant (Figures S1A, B). When luciferase (Luc)-MSCs were injected along with AAV1/IL-10 or AAV1/LacZ control vectors, which were also not transduced into the cells, a significant difference was observed in cell survival in the Luc-MSC-treated mice (Figure S1C, D). IL-10 plasmid-transfected Luc-MSCs expressed higher levels of IL-10 and were more effective at enhancing post-transplantation retention (Figure S2). We also confirmed a maximum 5.03-fold stronger luciferase signal intensity in muscles transplanted with IL-10-expressing MSCs than in control MSC-treated muscles at day 7 (See also Figure S2C), although most of these cells disappeared within 12 days. We developed a continuous IL-10 expression system of MSCs using an AAV vector to confirm the expected high and prolonged cell survival rates following transplantation. The IL-10-Luc-MSCs displayed a higher survival rate immediately after administration (luciferase signal at 3 days after transplantation, 4.41 ± 1.78 × 10^5^ counts), with the maximum value of 6.5-fold observed at 24 days (3.46 ± 1.12 × 10^4^ counts) compared to the signal corresponding to GFP-Luc-MSCs (2.98 ± 1.04 × 10^5^ counts, *P <* 0.0001; 5.35 ± 2.93 × 10^3^ counts, *<* 0.005*,* respectively), as observed in vivo in imaging (Fig. 1a*,* b) and immunohistological analyses (Figure S3). Significantly higher levels of IL-10 expression from AAV1/IL-10-transduced Luc-MSCs (IL-10-Luc-MSCs) were confirmed in vitro (Fig. 1c) and in treated muscles (See also Figure S4). The findings suggested that the higher retention in the early stage exerted a significant effect on long-term engraftment. Notably, IL-10-Luc-MSCs were retained for more than 67 days after transplantation (Fig. 1a, b).

### Successful long-term engraftment of IL-10-MSCs in injured muscle tissue

**Fig. 2:**
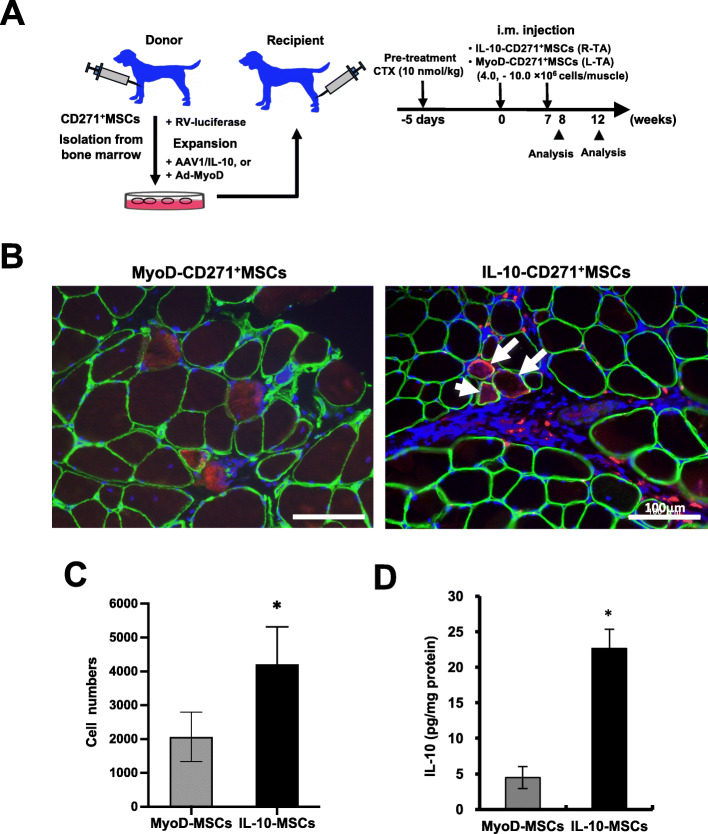
Successful long-term engraftment of canine IL-10-MSCs in the skeletal muscles of dogs. **a** Transplantation schedule. Canine CD271^+^MSCs expressing luciferase (Luc-CD271^+^MSCs) were transduced with AAV1/IL-10 in the cardiotoxin-pretreated tibialis anterior (TA) muscle of the recipient dog. **b** Immunofluorescence analysis of the TA muscle derived from a MyoD-Luc-CD271^+^MSC- (left panel) and IL-10-Luc-CD271^+^MSC-treated dog (5601MN, right panels) 8 weeks after injection using antibodies specific for luciferase (red), dystrophin (green), and nuclear stain 4′,6′-diamidino-2-phenylindole (DAPI, blue). Arrow, luciferase-positive myofibers. Bar = 200 μm. **c** Luciferase assays to determine the cell number in the IL-10-Luc-CD271^+^MSC- (IL-10-MSCs) or MyoD-Luc-CD271^+^MSC (MyoD-MSC)-treated TA muscle lysate. The mean was the average value of three measurements from each group. **d** Quantitative measurement of IL-10 levels in the IL-10-MSC- or MyoD-MSC-treated TA muscle lysate (mg protein) using ELISA. Data are presented as mean ± SD, and statistical differences between values for MyoD-MSC- and IL-10-MSC-treated TA muscle are indicated, ^*^P < 0.05, *t*-test

We also investigated the effects of IL-10 overexpression in CD271^+^MSCs derived from dog bone marrow using Beagle dogs as a larger animal model (Fig. 2a, Table 1). The high efficiency of adenovirus and AAV1 transduction in CD271^+^MSCs was confirmed based on immunofluorescence analysis or GFP signals (See also Figure S5)*.* CD271^+^MSCs transduced with AAV1/IL-10 (IL-10-Luc-CD271^+^MSCs) exhibited IL-10 overexpression in the culture medium (MyoD-MSCs, 3.1 pg/mL; IL-10-MSCs, 93.6 pg/mL). Four weeks after intramuscular injection, the accumulation of IL-10-Luc-CD271^+^MSCs was observed in the immunochemical analysis of cardiotoxin-injured tibialis anterior (TA) muscle tissues. Immunofluorescence analysis revealed the accumulation of luciferase-positive IL-10-Luc-CD271^+^MSCs around the inflammatory region in MSC-treated TA muscle 4 weeks after the secondary injection, comparable to the observation for MyoD-MSCs (Fig. 2b). Luciferase-positive muscle-like tissue was detected in the IL-10-Luc-CD271^+^MSC-treated muscle, similar to that observed in the muscle after treatment with Luc-CD271^+^ MSCs transduced with MyoD (MyoD-Luc-CD271^+^MSC), which is a key factor for myogenic determination, as described in a previous report [27]. These findings suggest that the Luc-CD271^+^MSCs transduced with AAV1/IL-10 formed myofibers. Luciferase activity, which correlated with the number of MSCs, tended to be higher in IL-10-Luc-CD271^+^MSC-treated whole TA muscles than in MyoD-Luc-CD271^+^MSC-treated muscle (Fig. 2c). The IL-10 levels in IL-10-Luc-CD271^+^MSC-treated TA muscles increased, while those in MyoD-Luc-CD271^+^MSC-treated muscles did not (Fig. 2d). These data suggest that IL-10-expressing CD271^+^MSCs could survive long-term and engraft after intramuscular injection during muscle regeneration.

### Safety and efficacy of systemic transplantation of IL-10-DPSCs in the DMD model

**Fig. 3:**
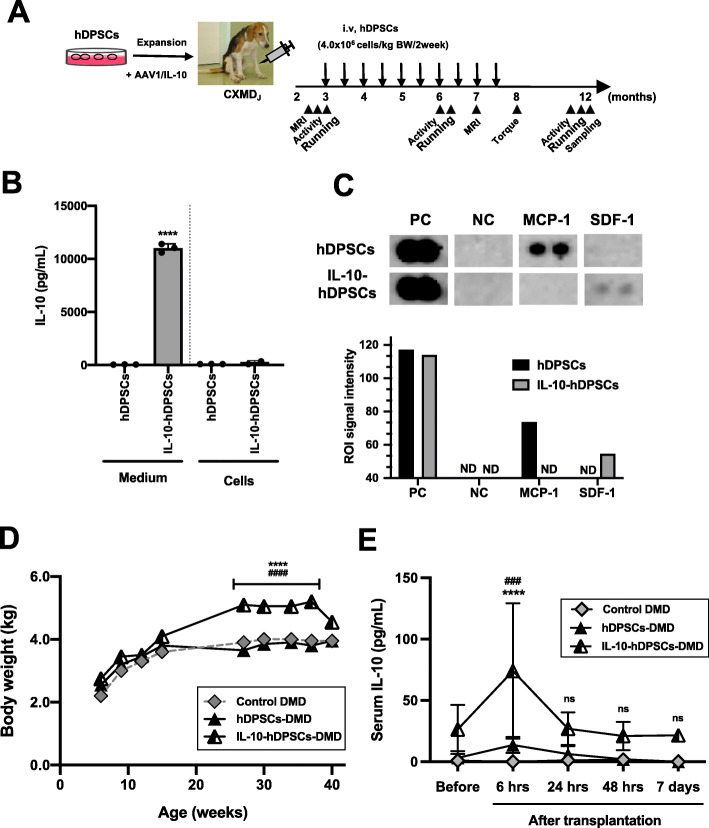
Safe systemic treatment with IL-10-expressing hDPSCs in the CXMD_J_ model. **a** Transplantation schedule. **b** Quantitative measurement of IL-10 expression in 2-day culture medium and in the hDPSC lysate (100 mg protein) using ELISA. Data are presented as mean ± SD, and statistical differences between hDPSCs vs. IL-10-hDPSCs are indicated (^****^P < 0.001, *n* = 3). **c** Cytokine and chemokine expression in 2-day culture media of hDPSCs and IL-10-hDPSCs analyzed using the Proteome ProfilerTM Array. Changes in the expression levels of monocyte chemotactic protein-1 (MCP-1), and stromal-derived factor-1 (SDF-1/CXCL12), compared to the positive control (PC) signals or negative control (NC). Signal intensity in the regions of interest (ROIs) quantified using array images (upper panels) and representative data (graph) are presented. ND, not detected. **d** Growth curve of untreated CXMD_J_ (control DMD; 14102MA), hDPSC-, and IL-10-hDPSC-treated CXMD_J_ (hDPSC-DMD, 14105MA; IL-10-hDPSC-DMD, 14108MA) dogs. Data are presented as mean ± SD, and statistical differences between control DMD vs. IL-10-hDPSC-DMD (^****^P < 0.001), hDPSC-DMD vs. IL-10-hDPSC-DMD (^####^*P <* 0.001) are indicated; one-way ANOVA. **e** Serum levels of IL-10 at 6, 24, and 48 h, and 7 days after transplantation (*n* = 3), quantified using ELISA. Data are presented as mean ± SD, and statistical differences between control DMD vs. IL-10-hDPSC-DMD (^****^P < 0.0001), hDPSC-DMD vs. IL-10-hDPSC-DMD (^###^*P <* 0.001, ^####^*P <* 0.0001) are indicated; ns, not significant, two-way ANOVA

Next, we evaluated the efficacy of IL-10-expressing MSCs by performing systemic transplantation using hDPSCs (Fig. 3a). We confirmed that the extracellular secretion of IL-10 from AAV1/IL-10-transduced-human DPSCs (IL-10-hDPSCs) significantly inhibited the expression of pro-inflammatory monocyte chemotactic protein-1 (MCP-1), and conversely upregulated stromal-derived factor-1 (SDF-1) in IL-10-hDPSCs compared with the levels observed in hDPSCs (Fig. 3b, c). IL-10-hDPSCs or hDPSCs were intravenously injected nine times at biweekly intervals in the acute phase in the CXMD_J_ model with the DMD phenotype (Table 1). No obvious abnormalities related to liver damage, kidney damage, or anemia were induced in response to the systemic administration of hDPSCs in the CXMD_J_ model. Transient increases in ALT, AST, and BUN levels were observed occasionally in the CXMD_J_ model, independent of treatment with hDPSCs (See also Figure S6). During the experiment, the IL-10-hDPSC-treated CXMD_J_ showed better growth compared to the untreated littermates, CXMD_J_ (*P <* 0.0001), and hDPSC-treated CXMD_J_ (*<* 0.0001), in terms of body weight (Fig. 3d). Although the serum levels of IL-10 increased transiently 6 h after IL-10-hDPSC injection (104.0 pg/mL vs. control DMD, vs. hDPSC-DMD, *<* 0.0001), the levels decreased rapidly within 24 h of transplantation (32.6 pg/mL vs. control DMD, *<* 0.0067; vs. hDPSC-DMD, *<* 0.0278), and did not differ significantly from the control CXMD_J_ 7 days after injection (21.6 pg/mL) (Fig. 3e). The levels of IL-6 in blood increased transiently in CXMD_J_ (maximum 742.9 pg/mL). In contrast, the levels in IL-10-hDPSC-treated CXMD_J_ were within the normal range (0–9.85 pg/mL) during the experiments. High levels of IFN-γ were also observed in CXMD_J_ (maximum 103.9 pg/mL) and hDPSC-treated CXMD_J_ (114.8 pg/mL), whereas the levels tended to be marginally lower in the IL-10-hDPSC-treated CXMD_J_ (68.4 pg/mL). The trend of cell retention in blood was similar for hDPSCs (17.9 ± 19.5 pg/100 ng genomic DNA) and IL-10-hDPSCs 24 h after transplantation (79.4 ± 7.89 pg/100 ng genomic DNA), as revealed by human-specific *Alu*-PCR. Long-term engraftment in tissues was investigated. hDPSCs were not detected in the skeletal muscle, lung, or liver tissues of the hDPSC-treated CXMD_J_. The IL-10-hDPSCs only survived and maintained the engraftment status 4 months after treatment in the TA muscle (56.1 pg/100 ng genomic DNA), whereas the cells were not detected in other organs. Conversely, dystrophin expression was undetectable in the muscle tissues of hDPSC-treated CXMD_J_ (Figure S7A).

### Morphological improvement in IL-10-hDPSC-treated dog with DMD

**Fig. 4:**
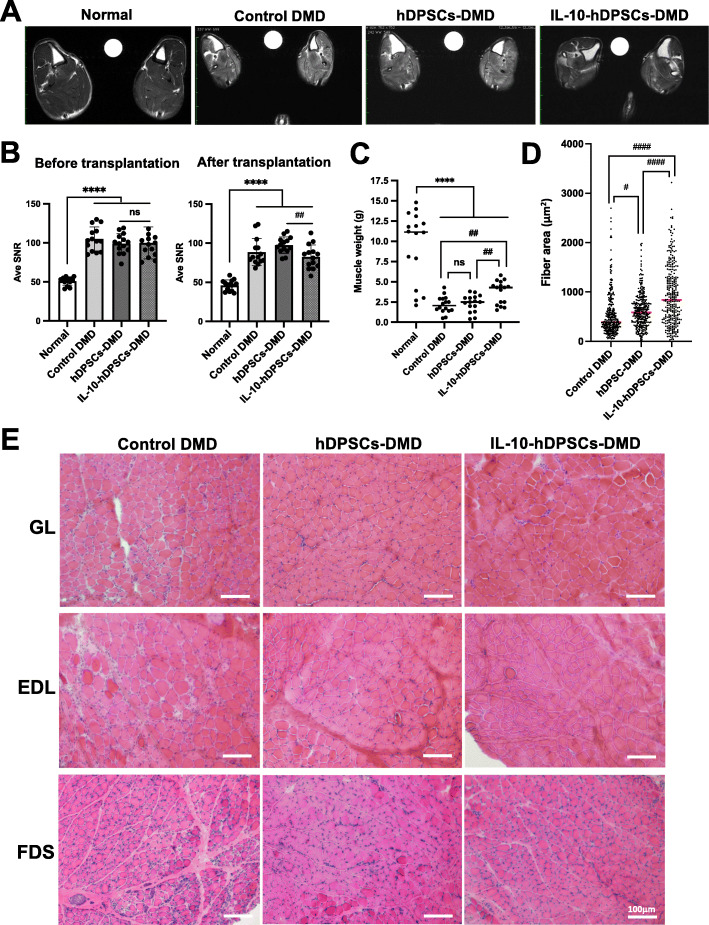
Improvement in the hDPSC-treated CXMD_J_ model observed by histological examination. **a** Cross-sectional magnetic resonance images (MRI) in the lower leg muscles of a 7-month-old normal dog, untreated CXMD_J_ (control DMD), hDPSC-treated (hDPSC-DMD), and IL-10-hDPSC-treated CXMD_J_ (IL-10-hDPSC-DMD). Muscle necrosis and inflammation based on the sequence of T2-weighted imaging of the lower legs for each dog were comparable (R, right side; L, left side, left/right asymmetry). **b** The averaged signal-to-noise ratios (Ave SNR) were calculated in the regions of interest (ROIs) from all muscles (*n* = 14) derived from each hindlimb of 2-month-old (before transplantation) and 7-month-old dogs (after transplantation). Data are presented as mean ± SD, and statistical differences between normal vs. control DMD (^****^P < 0.0001), hDPSC-DMD vs. IL-10-hDPSC-DMD (^##^*P <* 0.01) are indicated; ns, not significant, one-way ANOVA. **c** Weight of skeletal muscle (*n* = 16) from 1-year-old normal dog, untreated CXMD_J_ (control DMD), hDPSC-treated (hDPSC-DMD), and IL-10-hDPSC-treated CXMD_J_ (IL-10-hDPSC-DMD) shown as dot plots. Median values are presented as black bars. **d** Muscle fiber areas (μm^2^) measured from the extensor digitorum longus (EDL) muscle of 1-year-old control DMD, hDPSC-DMD, and IL-10-hDPSC-DMD dogs by hematoxylin and eosin (H&E) staining. Each fiber area is indicated by a dot, and the average fiber area is described as a red bar for each muscle. In total, 305 fibers are represented in the dot plot. Median values are indicated by the red bars. Statistical differences between normal vs. control DMD (^****^P < 0.0001), control DMD, or hDPSC-DMD vs. IL-10-hDPSC-DMD (^#^*P <* 0.05, ^####^*P <* 0.0001) are indicated; ns, not significant, one-way ANOVA. **e** H&E staining of the gastrocnemius lateral head (GL), EDL, and flexor digitorum superficialis (FDS) muscle from 1-year-old control DMD, hDPSC-DMD, and IL-10-hDPSC-DMD dogs. Scale bar, 100 μm

The high-intensity T2-signals observed in MRI, which were detected in the necrotic/edematous and inflammatory lesions in the dystrophic muscle, were significantly reduced in the cross-sectional muscles of the IL-10-hDPSC-treated dog (82.5 ± 16.9 average signal-to-noise ratios, SNRs) compared to the signals in hDPSC-treated CXMD_J_ (97.4 ± 13.3, *P* = 0.008) after transplantation (Fig. 4a, b). The weight of each muscle obtained from IL-10-hDPSC-treated CXMD_J_ (3.73 ± 1.43 g) increased compared to that in the untreated (2.16 ± 1.04 g, vs. IL-10-hDPSC- CXMD_J_, *<* 0.005) and hDPSC-treated CXMD_J_ (2.33 ± 1.04 g, *<* 0.005) (Fig. 4c). We also confirmed that the area of the muscle fiber in the extensor digitorum longus (EDL) muscle from IL-10-hDPSC-treated CXMD_J_ (935.7 ± 601.9 μm^2^) increased compared to that in the untreated (537.6 ± 439.2 μm^2^ vs. IL-10-hDPSC-CXMD_J_, < 0.0001) and hDPSC-treated CXMD_J_ (629.3 ± 352.8 μm^2^, < 0.0001) (Fig. 4d). The gastrocnemius lateral (GL), EDL, and flexor digitorum superficialis (FDS) muscles from CXMD_J_ displayed smaller (regenerating fibers) and larger (hypertrophic fibers) fiber diameter in the dystrophic muscles, spread muscle interstitium, and cell infiltration interspersed in the muscle interstitium (Fig. 4e). In contrast, the histopathological observations of the hDPSC- and IL-10-hDPSC-treated CXMD_J_ muscles revealed significantly limited infiltration of nuclei, which indicated a milder phenotype compared to untreated CXMD_J_. These data suggest that the repeated systemic administration of IL-10-hDPSCs induces morphological improvement, including inflammation regulation, in CXMD_J_. In addition, we performed quantitative analysis of collagen type III expression at 8 muscle tissue sites to investigate fibrosis. However, we could not confirm a significant difference among the groups (control DMD, 2.27 ± 2.18 pg/μg protein; hDPSC- and IL-10-hDPSC-DMD, 3.91 ± 1.66, and 6.17 ± 2.50 pg/μg protein, respectively, *P* = 0.317).

### Long-term maintenance of muscle function in IL-10-hDPSC-treated DMD dog

The CXMD_J_ developed progressive general weakness owing to the reduced strength of the skeletal muscles. The tetanic force in the hind limbs in CXMD_J_ (2.55 ± 0.42 N m/s) was 41.2 ± 5.1% of that in normal dogs (*P* < 0.0001) (Figure S8). Conversely, significantly higher torque values were observed in the IL-10-hDPSC-treated CXMD_J_ (4.17 ± 1.28 N m/s, 66.5 ± 12.2% of the value in the normal dog, < 0.0001) than in CXMD_J_ (< 0.0008), similar to that in hDPSC-treated CXMD_J_ (3.68 ± 0.57 N m/s). These results suggest that the progressive loss of limb muscle strength is ameliorated upon treatment with hDPSCs and IL-10-hDPSCs.

**Fig. 5:**
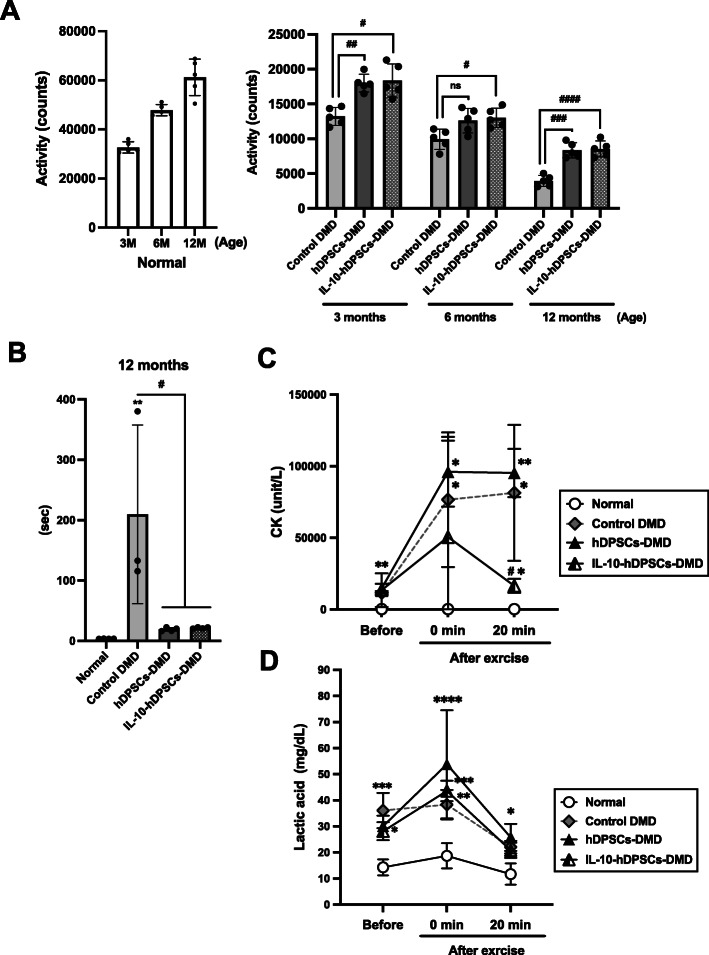
Improved locomotor activity in CXMD_J_ treated with IL-10-expressing MSCs. **a** Day-time behavioral activity of normal (left graph), untreated CXMD_J_ (control DMD), hDPSC-treated CXMD_J_ (hDPSC-DMD), and IL-10-hDPSC-treated CXMD_J_ (IL-10-hDPSC-DMD) in home cage at 3, 6, and 12 months presented as mean count activity (average of value for 5 days). Statistical differences between control DMD vs. hDPSC-DMD or IL-10-hDPSC-DMD dog groups (^#^*P <* 0.05, ^##^*P <* 0.01, ^###^*P <* 0.001, ^####^*P <* 0.0001) are indicated; ns, not significant, two-way ANOVA. **b** 15-m running speed of normal, control DMD, hDPSC-DMD, and IL-10-hDPSC-DMD dogs at 12 months. The mean value was the average value of four measurements from each group. Statistical differences between normal vs. DMD (^*^P < 0.05, ^**^P < 0.01, and ^****^P < 0.0001), control DMD vs. hDPSC-DMD, or IL-10-hDPSC-DMD (^#^*P <* 0.05, ^##^*P <* 0.01, ^###^*P <* 0.001) are indicated; ns, not significant, one-way ANOVA. Serum creatine kinase (CK) levels **c** and serum lactic acid levels **d** in each group before, 0, and 20 min after running exercise, which were four times greater than those after 15-m running, as measured using ELISA. Statistical differences between normal vs. DMD (^*^P < 0.05, ^**^P < 0.01), control DMD vs. IL-10-hDPSC-DMD (^#^*P <* 0.05) are indicated; two-way ANOVA. *n* = 4 for each group. Data are presented as the mean ± SD

Additionally, the physical activity of CXMD_J_ in the home cage also reduced drastically with age compared to that in normal dogs (Fig. 5a) [31]. Improved activity was confirmed in both groups, the hDPSC-treated (8340.4 ± 1090.3 counts; vs. control DMD, *P* = 0.0006) and IL-10-hDPSC-treated CXMD_J_ (8531.6 ± 1146.5 counts; vs. control DMD, < 0.0001), which was observed upon the comparison with 1-year-old CXMD_J_ littermates advanced symptoms (3954.4 ± 792.0 counts) (Fig. 5a). The IL-10-hDPSC-treated CXMD_J_ exhibited persistent and predominantly higher activity (6 months; 13,008.8 ± 1367.1 counts) than CXMD_J_ (9926.0 ± 1436.8 counts, < 0.005) as well as hDPSC-treated CXMD_J_ (12,605.8 ± 1756.3 counts). Furthermore, hDPSC- or IL-10-hDPSC-treated CXMD_J_ maintained a 15-m running speed and were active at 3 to 12 months of age (Fig. 5b, see also Figure S9, and Table S1; normal vs. control DMD, *P* < 0.0001–0.0062; control DMD vs. hDPSC-DMD, < 0.005; control DMD vs. IL-10-hDPSC-DMD, < 0.005). There was no significant difference in the running speed between hDPSC- and IL-10-hDPSC-treated CXMD_J_. However, the increased serum CK levels after running exercise (50,595 ± 67,255 unit/L) were restored immediately until 20 min in IL-10-hDPSC-treated CXMD_J_ (Fig. 5c, 16,490 ± 4850 unit/L; vs. hDPSC-treated DMD, *P* = 0.0134). Conversely, a persistent and significant increase in the serum CK levels was observed in hDPSC-treated CXMD_J_ (0 min, 96,075 ± 24,311 unit/L; 20 min, 95,300 ± 16,835 unit/L) as well as in the untreated CXMD_J_ (0 min, 76,650 ± 46,995 unit/L; 20 min, 81,425 ± 47,458 unit/L, *P* = 0.9277) after exercise. CXMD_J_ also showed higher concentrations of lactic acid before and after exercise compared to normal dogs. However, no significant change in the levels of lactic acid was observed in hDPSC-treated CXMD_J_ (Fig. 5d). These findings suggest that IL-10-hDPSCs exert a protective effect against dystrophic damage caused by exercise.

Overall, we observed that IL-10-expressing hDPSCs were able to ameliorate the dystrophic phenotype upon systemic repeated administration in dogs with DMD.

## Discussion

To improve the functionality of MSCs as a cell source, we focused on the overexpression of IL-10 based on its anti-apoptotic and anti-inflammatory properties. In this study, bone marrow MSCs derived from rat and CD271^+^ MSCs isolated from healthy dogs were injected intramuscularly into NOD/SCID mice or healthy Beagle dogs to study cell survival and engraftment in injured muscles (Figs. 1 and 2). hDPSCs exhibit cellular properties that are highly similar to those of bone marrow MSCs. Human-origin DPSCs or IL-10-transduced hDPSCs were also administered via intravenous injection into CXMD_J_ to investigate their systemic therapeutic effects (Figs. 3, 4, and 5). Our results indicate an improvement in survival rate, engraftment, and protective effects of different types of MSC in muscle tissues. This property is considered to be highly common to MSCs.

The higher survival rate of IL-10-MSCs in the early stage immediately after transplantation, as observed in the in vivo imaging analysis, is thought to facilitate long-term cell retention (Fig. 1), suggesting that stable IL-10 expression enabled the long-term survival and engraftment of MSCs after transplantation. Our findings suggest that during skeletal muscle regeneration, prolonged engraftment of IL-10-expressing CD271^+^MSCs eased the formation of new myofiber-like tissue and preservation of a functional contractile apparatus, following exposure to the muscle stem cell niche/microenvironment (Fig. 2). Rarely, MSCs differentiate into myogenic lineage cells in the absence of triggers such as MyoD (See also Figure S5D, E) [34], 5-azacytidine [35], and Notch I intracellular domain [4]. Although we could not confirm the myogenic differentiation of IL-10-MSCs in this study, as shown in Figure S7B, IL-10 is also considered to play a role in the long-term engraftment and survival of MSCs in muscle tissue, resulting from their association with surrounding myogenic stem/progenitor cell populations, such as satellite cells, skeletal muscle-MSCs, fibro-adipogenic progenitors (FAB), and myo-endothelial progenitors [36] for muscle repair. Furthermore, treatment of myocardial infarction with CD271^+^ bone marrow MSCs inhibited the expression of inflammatory cytokines and significantly upregulated pro-angiogenic VEGF [37]. CD271^+^ MSCs expressing IL-10 are also thought to enhance pro-angiogenic differentiation in injured tissue as a result of prolonged survival.

It was reasoned that IL-10-expressing hDPSCs exerted enhanced anti-inflammatory and protective effects on damaged tissue owing to the downregulation of MCP-1 and upregulation of SDF-1 in IL-10-hDPSCs (Fig. 3). SDF-1 is a crucial factor that supports tissue regeneration, and the roles of SDF-1 in MSC paracrine-mediated tissue repair have been reported [38]. Furthermore, it was also reported that the expression of multiple pro-angiogenic factors, such as SDF-1, FGF-2, IGF-1, and VEGF-A, is upregulated in IL-10-MSC-treated cardiac muscle [19], suggesting changes in the surrounding microenvironment.

In the dog model, the repeated systemic transplantation of hDPSCs and IL-10-expressing hDPSCs was safe and effective as a strategy for DMD therapy, as indicated by the blood tests, growth, spontaneous activity, and running function (Figs. 3 and 4). Long-term engraftment of hDPSCs was only confirmed in the dystrophic muscles in IL-10-DPSC-treated DMD, which suggests that hDPSC engraftment was enhanced in response to IL-10 paracrine effects. For example, SDF-1 and growth factors might enhance DPSC retention by altering the microenvironment. SDF-1 and its receptor CXCR4 and CXCR7 stimulate the production of paracrine mediators, including VEGF, β-FGF-1, and HGF, which exert anti-apoptotic, pro-angiogenic, and anti-inflammatory effects [39]. In addition, it is revealed that muscle regeneration is associated with muscular re-expression of CXCR4 in dystrophic muscle [40]. Based on this, we consider that IL-10-DPSC-specific engraftment and therapeutic effects may be associated with DMD treatment.

These facts provide evidence of the accumulation of hDPSCs at the site of inflammation after systemic administration, similar to that of MSCs. The functional recovery in the dystrophic skeletal muscles was attributed to the alleviation of the morphological pathologies, as indicated by the MRI findings and the histopathological appearance of samples from the hDPSC- and IL-10-hDPSC-treated CXMD_J_; however, fibrosis was not prevented (Fig. 4). Indeed, both hDPSC- and IL-10-hDPSC-treated CXMD_J_ showed improved limb strength, as evidenced by the tetanic force, revealing an improvement in spontaneous activity and running speed, while no significant difference was observed in the treated CXMD_J_ while maintaining apparent function. We observed that the IL-10-hDPSC-treated CXMD_J_ was more stable than the hDPSC-treated CXMD_J_ because the increase in serum CK levels after exercise was rapidly stabilized in the IL-10-hDPSC-treated CXMD_J_ (Fig. 5c). These facts suggest that treatment with IL-10-hDPSCs provides protection from physical damage-induced muscle injury in CXMD_J_, as opposed to the effect observed in dogs injected with untreated or non-transgenic cells, which is further evidenced by the effects of the modified characteristics of IL-10-hDPSCs involved in SDF-1 and VEGF. However, it was not clear whether the protective effects of IL-10-hDPSCs on the dystrophic muscle were caused by IL-10 directly or indirectly. The molecular mechanisms underlying the action of IL-10-hDPSCs are expected to be elucidated in future studies.

This is the first report of increased cell survival, engraftment, and possible tissue formation induced by IL-10-secreting MSCs in muscle tissues. In our previous report, myogenic lineage-MSCs were successfully engrafted in muscle tissues [27]. However, a more efficient transplantation strategy is required for functional improvement of muscle dystrophy. This study evaluated the possibility of improving survival, engraftment, and immune modulation of MSCs by AAV vector-mediated stable expression of IL-10.

We have previously provided evidence that severe phenotypes in IL-10 knockout *mdx* mice, such as increased M1 macrophage infiltration, high inflammatory factor levels, and progressive cardiorespiratory dysfunction, show a predisposition toward inflammation [41]. Glucocorticoids are widely used in patients to interrupt and improve muscle strength during early stages, which may also act directly on muscle fibers by stabilizing sarcolemma [42, 43]; however, this method is frequently associated with severe side effects. In our strategy, MSCs appeared to exhibit inflammatory regulation effects and a protective effect in the dystrophic muscle through the suppression of M1 macrophage infiltration by secreting IL-10. In addition, our stable IL-10 expression system was found to be safe, with a low risk of genome insertion owing to the use of an AAV vector. Random integration, off-target effects, and poor specificity are associated with the use of other viruses and genome engineering techniques. IL-10-expressing MSCs are expected to have potential applicability in muscle regeneration and treatment of muscle diseases. We have previously reported that IL-10 overexpression promotes neuroprotection in an experimental acute ischemic stroke model [44]. There is clinical interest in the applicability of IL-10-MSCs in ex vivo cell therapy owing to their anti-inflammatory properties and ability to release cytokines into the surrounding environment, which mediate their paracrine effects and modify the developmental fate of neighboring cells. We expect that the multiple characteristics and regenerative effects of MSCs alone as well as in combination with CD271^+^ IL-10 MSCs will result in an improved therapeutic impact in DMD.

## Conclusions

Our methods were developed to enhance MSC survival and improve their therapeutic effects using the anti-inflammatory properties of IL-10 in DMD treatment. In case of local injection, the IL-10-MSCs could maintain the long-term engraftment status and facilitate associated tissue repair. In case of repeated systemic administration, the IL-10-MSCs also protected the muscles from physical damage-induced injury, which improved the signs of muscle dysfunction in DMD. We can conclude that the local and systemic administration of IL-10-MSCs may exert beneficial IL-10 paracrine effects, which have potential value in DMD therapeutics.

## Supplementary Information

**Additional file 1: Figure S1**. In vivo bioluminescence imaging for the detection of injected MSCs. **Figure S2**. Enhanced engraftment of IL-10-MSCs. **Figure S3**. MSC survival in skeletal muscle tissue. **Figure S4**. IL-10 expression in IL-10-MSC-treated muscle tissue. **Figure S5**. Characterization of gene-transduced MSCs. **Figure S6**. Serum chemistry data from the hDPSC-treated CXMD_J_ model. **Figure S7**. Reverse transcription PCR for evaluating human specific dystrophin expression. **Figure S8**. Estimated isometric tetanic force in hDPSC-treated CXMD_J._ **Figure S9**. 15-m running speed of hDPSC-treated CXMD_J_. **Table S1**. Running speed (15-m) of dogs aged 12 to 44 months

## Data Availability

The datasets used and/or analyzed during the current study are available from the corresponding author on reasonable request.

## Abbreviations

AAV: Adeno-associated virus
ALT: Alanine aminotransferase
BSA: Bovine serum albumin
BUN: Blood urea nitrogen
CD: Cluster of differentiation
CK: Creatine kinase
CXMD_J_: Canine X-linked muscular dystrophy model in Japan
DAB: 3,3′-Diaminobenzidine
DAPI: 4′,6′-Diamidino-2-phenylindole
DMD: Duchenne muscular dystrophy
DMEM: Dulbecco’s modified Eagle’s medium
DPSCs: Dental pulp stem cells
eGFP: Enhanced green fluorescent protein
EDL: Extensor digitorum longus
FAB: Fibro-adipogenic progenitors
FBS: Fetal bovine serum
FDS: Flexor digitorum superficialis
GAPDH: Glyceraldehyde 3-phosphate dehydrogenase
GL: Gastrocnemius lateral
GVHD: Graft-versus-host disease
HLA: Human leukocyte antigen
HRP: Horseradish peroxidase
IFN-γ: Interferon-gamma
IL-10: Interleukin-10
iPS: Induced pluripotent stem cells
MCP-1: Monocyte chemotactic protein-1
MSCs: Multipotent mesenchymal stromal cells
MRI: Magnetic resonance imaging
PBS: Phosphate-buffered saline
PCR: Polymerase chain reaction
SD: Standard deviation
SDF-1: Stromal-derived factor-1
SNRs: Signal-to-noise ratios
TA: Tibialis anterior
TALEN: Transcription activator-like effector nuclease
TNF-α: Tumor necrosis factor-alpha
VEGF: Vascular endothelial growth factor
VSV-G: Vesicular stomatitis virus-glycoprotein
