# Supplementary material for: Enhanced cell survival and therapeutic benefits of IL-10-expressing multipotent mesenchymal stromal cells for muscular dystrophy
Source: Stem Cell Res Ther. 2021 Feb 4;12:105. doi: 10.1186/s13287-021-02168-1 (PMC7860619; doi:10.1186/s13287-021-02168-1)
Supplement: Supplementary file 1 — Additional file 1: Figure S1. In vivo bioluminescence imaging for the detection of injected MSCs. Figure S2. Enhanced engraftment of IL-10-MSCs. Figure S3. MSC survival in skeletal muscle tissue. Figure S4. IL-10 expression in IL-10-MSC-treated muscle tissue. Figure S5. Characterization of gene-transduced MSCs. Figure S6. Serum chemistry data from the hDPSC-treated CXMDJ model. Figure S7. Reverse transcription PCR for evaluating human specific dystrophin expression. Figure S8. Estimated isometric tetanic force in hDPSC-treated CXMDJ. Figure S9. 15-m running speed of hDPSC-treated CXMDJ. Table S1. Running speed (15-m) of dogs aged 12 to 44 months [file 13287_2021_2168_MOESM1_ESM.pdf]

Supplemental Methods

Cell preparation

To generate luciferase-expressing MSCs, MSCs isolated from Sprague-Dawley rat bone-marrow [1] were transduced with a vesicular stomatitis virus-glycoprotein (VSV-G)-pseudotyped retroviral vector encoding firefly luciferase [2]. Cells were maintained in DMEM supplemented with 10% FBS, 100 U/mL penicillin, and 100 μg/mL streptomycin (Sigma-Aldrich, St. Louis, MO, USA). For transplantation, the cells were washed with PBS to completely remove the culture medium containing vectors.

Transplantation in mice

NOD/SCID mice were purchased from Nihon CLEA (Tokyo, Japan) and were housed at the National Center of Neurology and Psychiatry (Tokyo, Japan). Luciferase-expressing rat MSCs were injected intramuscularly into the right or left hind-limb muscle of NOD/SCID mice pretreated (1 day before treatment) with cardiotoxin (10 μM, Merck KGaA, Darmstadt, Germany). Recombinant IL-10 (0.3,1.0 μg/site) was injected into the MSC-treated sites on days 0, 2, and 4 after MSC transplantation (Figures S1A, B). Luciferase-MSCs were injected concomitantly with AAV1/IL-10 vector (left site) or AAV1/LacZ vector (right site, 5–10 × 10^8 g, each) into the hind-limb muscle of NOD/SCID mice, immediately after a mixture of MSCs and AAV vector was injected (Figures S1C, D). Luciferase-expressing MSCs were transfected with an eGFP or IL-10 expression vector (pW-CAG-EW or pW-CAG-IL-10). IL-10 or eGFP expression vector-transfected Luc-MSCs (2.5 × 10^6 cells), AAV1/IL-10, or AAV1/eGFP vector-transduced Luc-MSCs (1.0 × 10^7 cells) were injected into the right (eGFP) and left (IL-10) hind-limb muscle of NOD/SCID mice (Figure S2).

In vivo imaging analysis

After the injection of luciferase expressing rat-MSCs on day 0 of the experiment, in vivo luminescence images were acquired periodically to assess the engraftment efficiency and cell survival in the transplanted mice. Prior to imaging, the mice were anesthetized by inhalation of 2.0% isoflurane and oxygen and injected intraperitoneally with 150 mg luciferin (Summit Pharmaceuticals International Corp., Tokyo, Japan.) per kg body weight. *In vivo* images were acquired using the IVIS charge-coupled-device camera system (Xenogen Corp., Alameda, CA) at multiple time points. The region of interest (ROI) luminescence signals from individual MSC-injected sites were measured using the Living Image® 3.2 software package (Xenogen Corp.).

ELISA

The IL-10 expression levels in the muscle lysate obtained from the MSC-treated animals were measured using the Quantikine ELISA mouse IL-10 Immunoassay (Thermo Fisher Scientific) according to the manufacturers' recommendations. The final values were normalized to the protein concentrations, which were measured using the Pierce® BCA Protein Assay Kit (Thermo Fisher Scientific).

Histopathological and immunohistochemical analyses

Samples from MSC-treated muscles were collected and immediately frozen in liquid nitrogen-cooled isopentane. Transverse cryosections 8 μm in thickness prepared from skeletal muscles were stained with H&E using standard procedures. For immunohistochemical analyses, thick cryosections were fixed in acetone for 5 min at –20 °C. The tissue sections were then blocked with 0.5% bovine serum albumin (BSA) in PBS. The following antibodies were used for antigen detection at 1:40–1:50 dilutions: rabbit anti-firefly luciferase (ab21176; Abcam Plc., Cambridge, UK). Cells cultured on glass cover slips (in 24-well plates) were fixed in 1% formaldehyde-PBS for 1 h at 4 °C, washed twice with PBS containing 10 mM glysin, and permeabilized in PBS containing 0.1% Triton X-100 for 5 min. The cell monolayers were incubated with 2% skim milk for blocking and washed twice with PBS and mouse anti-myogenin antibodies (1: 100) in 1% skim milk for 1 h at 20-25 °C. After washing with PBS, the antibodies were diluted using 0.5% BSA in PBS and used to treat the cells or tissue sections overnight at 4 °C. The tissue sections were washed with PBS and then probed with Alexa 568-conjugated anti-rabbit IgG antibodies (Thermo Fisher Scientific) and Alexa 488-conjugated anti-mouse IgG antibodies (Thermo Fisher Scientific) at 1:250–1:100 dilution for 1 h at 4°C. The coverslip slides were washed with PBS and mounted in Vectashield (Vector Laboratories Inc., Burlingame, CA, USA) with 4′,6′-diamidino-2-phenylindole (DAPI). Immunofluorescence analysis was performed using an IX71 fluorescence microscope (Olympus, Tokyo, Japan).

Reverse transcription polymerase chain reaction

Total RNA (2 μg) was isolated from muscle samples disrupted in a Multi-Beads Shocker (Yasui Kikai, Osaka, Japan) using the RNeasy Micro kit (Qiagen). First-strand cDNA was synthesized using the Super Script III First Strand Synthesis System for RT-PCR (Thermo Fisher Scientific). For each PCR assay, 500 ng of cDNA was used. The primers used in this study were as follows: human dystrophin forward, 5′-TGAAACTGGAGGACCCGTG-3′ (1st reaction), 5′- AAAAGACCTTGGGCAGCTTG-3′ (2nd reaction); reverse, 5′- CCAAGAGGCATTGATATTCTC-3′. As an internal control, a primer set for the housekeeping gene glyceraldehyde-3-phosphate dehydrogenase (*GAPDH*) was used as follows: mouse, forward, 5′- GATGACATCAAGAAGGTGGTGA-3’, and reverse, 5’-TGCTGTAGCCGTATTCATTGTC-3’; canine, forward, 5′-GCGAGATCCCGCCAACATCAAA-3′, and reverse, 5′- AGGAGCAGAGATGATGACCCTC-3′. Quantitative PCR was carried out using SYBR® Premix Ex TaqTM II (Perfect Real Time, Takara Bio Inc.) SYBR green detection of PCR products was conducted in real time using the MyiQ single-color detection system (Bio-Rad, Hercules, CA, USA). Wild type or mutation in dystrophin gene was confirmed using *mdx*-amplification-resistant mutation system PCR [3].

Blood test

The dogs underwent periodic veterinary examinations at 1–2 week intervals until sampling. Hematological and serum biochemical testing for creatine kinase (CK) was performed using a model F-820 semi-automated hematology analyzer (Sysmex, Hyogo, Japan). The levels of serum alanine aminotransferase (ALT), aspartate aminotransferase (AST) and blood urea nitrogen (BUN) were determined using a DRI-CHEM3506 automated analyzer (Fuji Film, Tokyo, Japan).

Hind-limb extensor strength test

The functional status of the two hind limbs in $CXMD_J$ was evaluated by measuring the flexion and extension strengths of the wrist using a customized torque measurement device. Stimulation frequencies from 60 Hz can activate muscles that extend or push the hind paw against the ground. In this instrument, a transducer captured the torque generated when the paw pushed against the force plate. The maximal torque was expressed as a percentage of predicted values computed using a model based on control values with respect to the animal weight. $P < 0.05$ was considered statistically significant.

Running activity

The physical activity levels of $CXMD_J$ and littermate normal dogs used as controls were monitored during the experimental period. We measured the 15-m running time of normal and $CXMD_J$ littermates during the experimental period. The running speed was averaged four times.

Supplemental figure legends

Figure S1. *In vivo* bioluminescence imaging for the detection of injected MSCs

(A) Luciferase-expressing MSCs (5.0×10^6 cells) with or without recombinant IL-10 were injected into the hind limb muscle of NOD/SCID mice (right site, IL-10 (-); left site, 0.3 or 1.0 μg of IL-10 (+)). *In vivo* bioluminescence imaging of luciferase signals from MSC-treated mice was conducted 2 and 4 days after injection. **(B)** Quantitative ratio of IL-10 (-) to IL-10 (+) luciferase signals from the MSC-injected sites (Day 4). n = 3 for each group, *t*-test. **(C)** Luciferase-expressing MSCs were injected immediately after a mixture with IL-10 (left site) or LacZ-expressing AAV1 vector (right site) was injected into the hind limb muscle of NOD/SCID mice (left panel, 5.0×10^8 genome copies (g.c.); right panel, 1.0×10^9 g.c.). *In vivo* bioluminescence imaging of MSC-treated mice showed luciferase signals at 9 days after injection. **(D)** The quantitative luciferase signals from the MSC-injected sites (right site, Lac Z; left site, IL-10) from imaging analysis. n = 4 for each group, *t*-test.

Figure S2. Enhanced engraftment of IL-10-MSCs

Luciferase-expressing MSCs were transfected with eGFP or IL-10 expression plasmid vectors (n = 3, $^*P < 0.05$). **(A)** Quantitative measurement of IL-10 expression levels in MSC culture using ELISA (n = 3). **(B)** eGFP- or IL-10-expressing MSCs were injected into the right or left hind limb muscle (eGFP-MSC, IL-10(-); or IL-10-MSCs, IL-10(+)) of NOD/SCID mice. *In vivo* bioluminescence imaging of the

MSC-treated mice shows the luciferase signals 0–12 days after injection. **(C)** Quantitative luciferase counts from the eGFP- or IL-10-expressing MSC-injected sites; IL-10 (-) and IL-10 (+) luciferase signals from imaging analysis at 3–12 days after injection. The graph indicates the relative change in signals on the day of injection as 1.0. The mean value was the average value for each group (n = 3–4). Statistical differences between GFP-MSCs vs. IL-10-MSCs ($^{*}P < 0.05$) are indicated, multiple *t*-test.

Figure S3. MSC survival in skeletal muscle tissue
The luciferase-expressing MSCs were transduced using control AAV1/enhanced green fluorescence protein (GFP) or AAV1/IL-10 vector. GFP- or IL-10-expressing MSCs were injected into the right or left hind-limb muscle of NOD/SCID mice. **(A)** Immunohistochemical analysis using horseradish peroxidase (HRP)-diaminobenzidene (DAB)-labeled luciferase antibodies was performed to observe of the treated muscle derived from mice (GFP-MSCs, and IL-10-MSCs). Circles with dotted lines (blue) indicate the accumulation of luciferase-immunopositive MSCs. Scale bar, 100 µm. **(B)** The quantitative luciferase counts at the site of MSC injection, IL-10 (-) and IL-10 (+) luciferase signals from imaging analysis during 12 days after injection. Data monitoring revealed the luciferase activity in each mouse. The mean value was the average value in each group (n = 3). Statistical differences between GFP-MSCs vs. IL-10-MSCs ($^{*}P < 0.05$) are indicated, *t*-test.

Figure S4. IL-10 expression in IL-10-MSC-treated muscle tissue
ELISA was performed for quantitatively measuring IL-10 expression in eGFP- or IL-10-expressing MSC (IL-10(-) or IL-10(+))-treated muscle at 4 and 9 days after injection. Quantitative IL-10 signals were correlated based on the quantity of protein (pg/µg protein).

Figure S5. Characterization of gene-transduced MSCs
(**A**) MSCs isolated from bone-marrow were transduced with vesicular stomatitis virus-glycoprotein-pseudotyped retroviral vector encoding firefly luciferase (Retro-luciferase). To evaluate luciferase expression, immunofluorescence analysis was performed using anti-luciferase antibody. Left panel, untransduced control (-); Right panel, transduced MSCs (+). Scale bar, 100 µm. (**B**) Canine CD271^{+}MSCs were transduced with adenovirus-GFP (Ad-GFP). Merged images show bright field and fluorescent signals detected from MSCs 12 days after transduction. Scale bar, 100 µm. (**C**) Canine CD271^{+}MSCs were transduced with AAV1/GFP. GFP signals and merged bright images of MSCs 8 days after transduction. Scale bar, 500 µm. (**D**) Skeletal muscle tissue was isolated from *mdx* mice at 8 weeks post-transplantation of MyoD-transduced (+) or untransduced (-) rat MSCs and were subjected to immunofluorescence analysis using anti-luciferase antibody. Scale bar, 100 µm. MyoD-transduced MSCs showed muscle fiber morphology, suggesting *in vivo* myogenic differentiation, whereas untransduced MSCs did not. (**E**) To assess *in vitro* myogenic differentiation, MyoD-transduced (+, right panel) or untransduced (-, left panel) canine CD271^{+}MSCs at 8 day of culture were subjected to immunofluorescence analysis using anti-myogenin antibody. Upper panels, myogenin signals; Lower panels, 4',6-diamidino-2-phenylindole (DAPI) signals. Scale bar, 100 µm. (**F**) GFP signals from hDPSCs transduced with AAV1/GFP 10 days after transduction (left panel), and cell morphology (right panel). Scale bar, 500 µm.

Figure S6. Serum chemistry data from the hDPSC-treated CXMD$_J$ model
Serum chemistry data were obtained from untreated CXMD$_J$ (control DMD), hDPSC-treated CXMD$_J$ (hDPSC-DMD), and IL-10-hDPSC-treated CXMD$_J$ (IL-10-hDPSCs-DMD) dogs at every point of administration throughout the experimental period; depicted here: serum alkaline phosphatase (ALP), aspartate transferase (AST), and blood urea nitrogen (BUN) levels.

Figure S7. Reverse transcription PCR for evaluating human-specific dystrophin expression
(**A**) To evaluate dystrophin expression in hDPSCs in skeletal muscle, RNA was isolated from human rhabdomysarcoma (RD) cells as control, the tibialis anterior (TA) muscle, extensor carpi ulnaris (ECU), gastrocnemius medial head (Gas) muscles, diaphragm and heart of untreated CXMD$_J$ (14102MA; control DMD), hDPSC-treated CXMD$_J$ (14105MA; hDPSC-DMD), IL-10-hDPSC-treated CXMD$_J$ (14108MA; IL-10-hDPSCs-DMD), and TA muscle from healthy dog (14103MN). Reverse transcription PCR was performed using canine-, or human-specific dystrophin primers. The PCR products were separated and analyzed using agarose-gel electrophoresis. (**B**) To determine the dystrophin expression in rat MSCs with AAV1/IL-10 in *mdx* mouse, RNA was isolated from the TA muscle of wild-type (WT, B6 mouse), *mdx*

mouse (control), and MSC-treated *mdx* mice with (+) or without (-) AAV1/IL-10 at 2 and 4 weeks after intra-muscular injection (mouse #1, 2, each). Reverse transcription PCR was performed using WT-, or mutant dystrophin-, or mouse *GAPDH*-specific primers. PCR products were separated and analyzed using agarose-gel electrophoresis; the arrow indicates specific positions.

Figure S8. Estimated isometric tetanic force in hDPSC-treated CXMD$_J$

Estimated isometric tetanic force in the hind limbs of 7-month-old normal dogs, untreated control DMD (n = 9), hDPSC-DMD (n = 8), and IL-10-hDPSC-DMD (n = 5) dogs. Supramaximal percutaneous stimulation of the sciatic nerve at the mid-femur induced the contraction of the right- and left site tibiotarsal joint extensors. The graph outlines the summary statistics of force change relative to baseline values 3 weeks after the final injection. Statistical differences between normal vs. control DMD ($^{****}P <$ 0.0001), control DMD vs. hDPSC-DMD, or IL-10-hDPSC-DMD ($^{\#\#}P < 0.01$, $^{\#\#\#}P < 0.001$) are indicated; ns, not significant, one-way ANOVA.

Figure S9. 15-m running speed of hDPSC-treated CXMD$_J$

15-m running speed of normal, control DMD, hDPSC-DMD, and IL-10-hDPSC-DMD dogs at 3, 6, and 12 months. The mean value was the average value of four measurements from each group. Statistical differences between normal vs. DMD ($^{*}P < 0.05$, $^{**}P < 0.01$, and $^{****}P < 0.0001$), control DMD vs. hDPSC-DMD, or IL-10-hDPSC-DMD ($^{\#}P < 0.05$, $^{\#\#}P < 0.01$, $^{\#\#\#}P < 0.001$) are indicated; ns, not significant, one-way ANOVA.

Supplemental Table 1. Running speed (15-m) of dogs aged 12 to 44 months

Age (weeks)	(sec)	Normal	Control DMD	hDPSCs-DMD	IL-10-hDPSCs-DMD
12	Mean	3.75	14.48	8.15	7.55
	SD	0.47	2.08	1.87	2.53
24	Mean	3.63	25.3	11.6	13.1
	SD	0.30	5.98	1.57	0.78
44	Mean	3.60	209.6	19.0	21.6
	SD	0.18	148.0	2.99	1.17

References

1. Uchibori R, Okada T, Ito T, Urabe M, Mizukami H, Kume A, and Ozawa K: **Retroviral vector-producing mesenchymal stem cells for targeted suicide cancer gene therapy.** *J Gene Med.* 2009, **11:** 373-381.
2. Nitahara-Kasahara Y, Hayashita-Kinoh H, Ohshima-Hosoyama S, Okada H, Wada-Maeda M, Nakamura A, Okada T, and Takeda S: **Long-term engraftment of multipotent mesenchymal stromal cells that differentiate to form myogenic cells in dogs with Duchenne muscular dystrophy.** *Mol Ther.* 2012, **20:** 168-177.
3. Amalfitano A, and Chamberlain JS: **The mdx-amplification-resistant mutation system assay, a simple and rapid polymerase chain reaction-based detection of the mdx allele.** *Muscle Nerve.* 1996, **19:** 1549-1553.

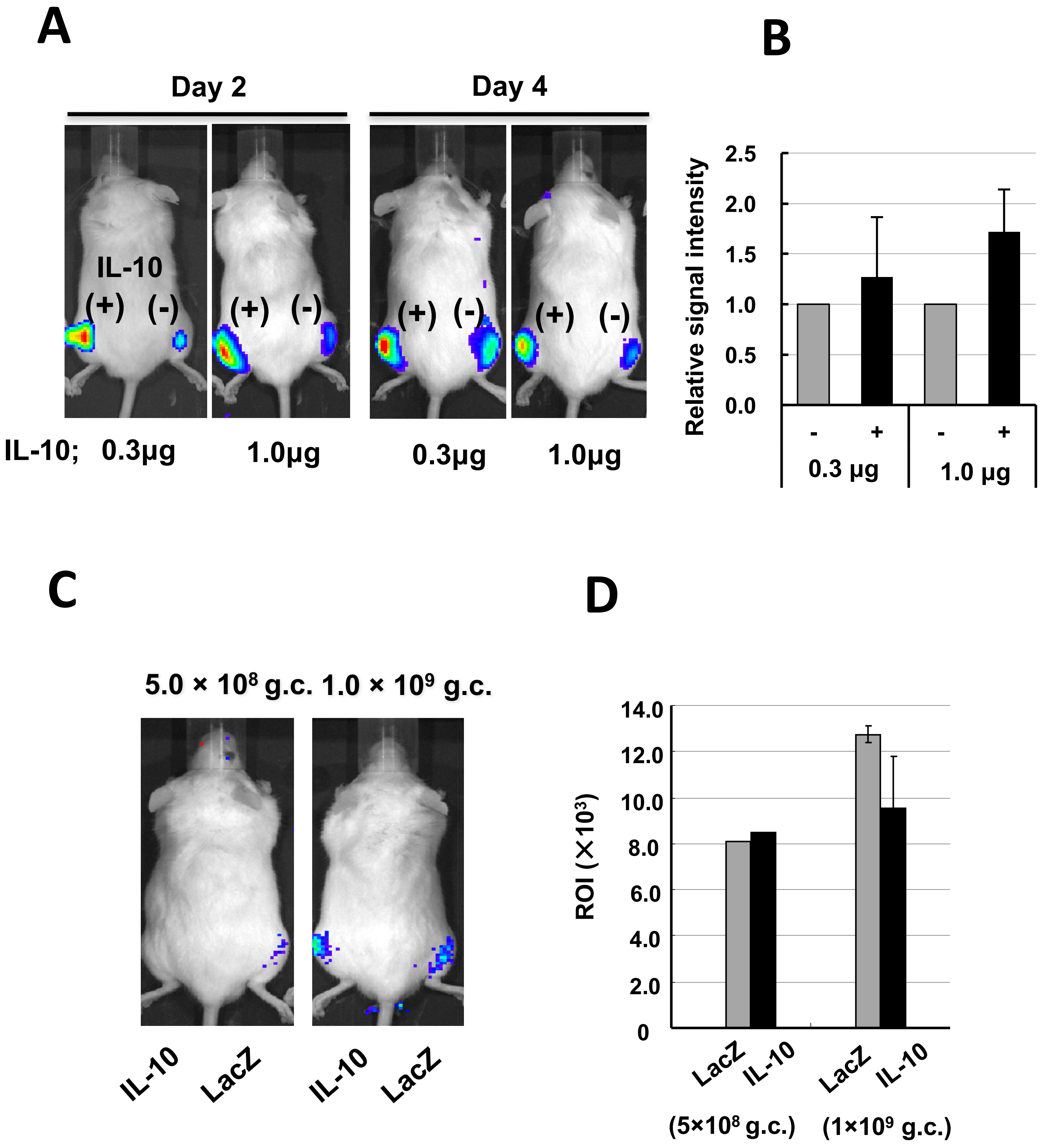

Supplementary Figure 1

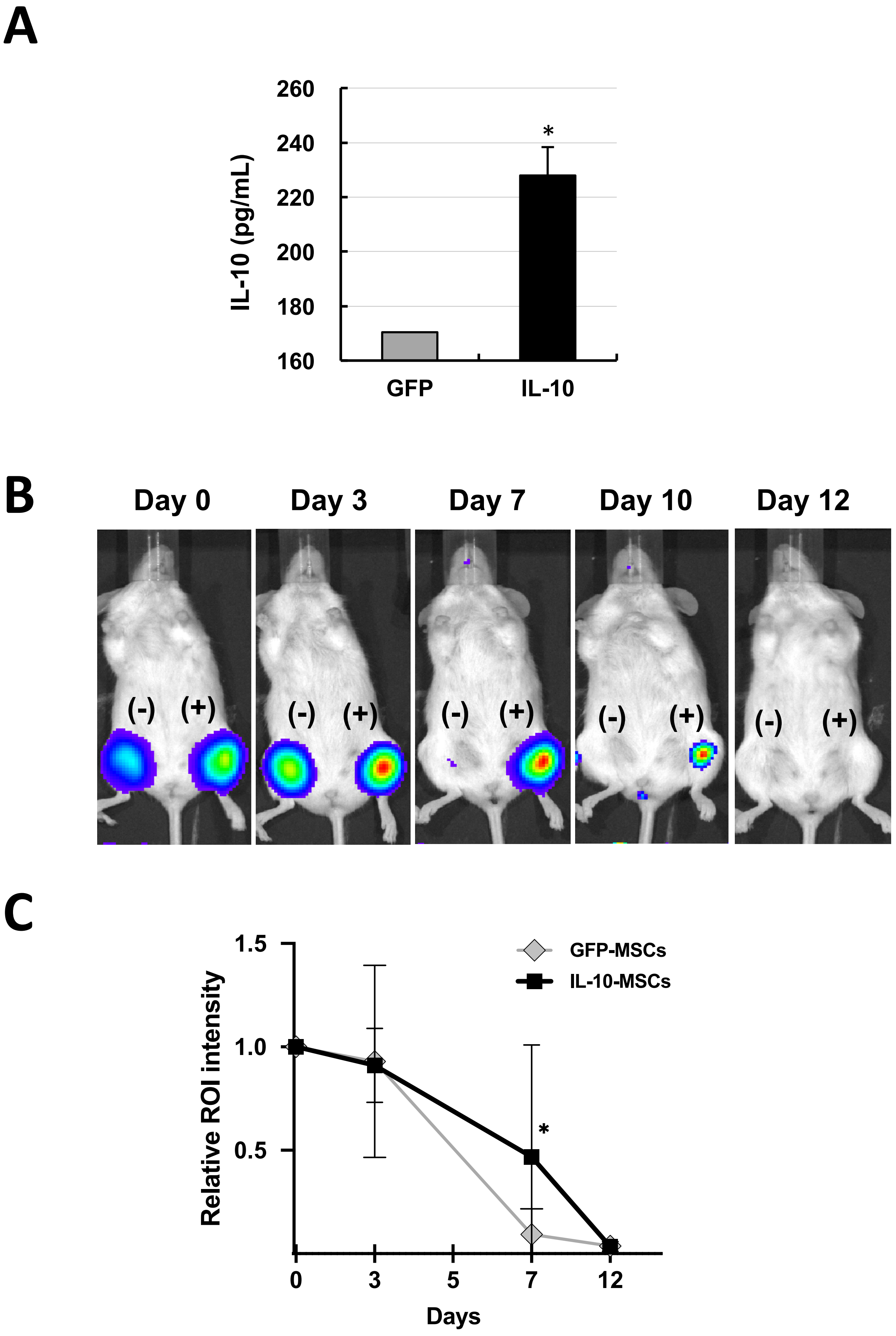

Supplementary Figure 2

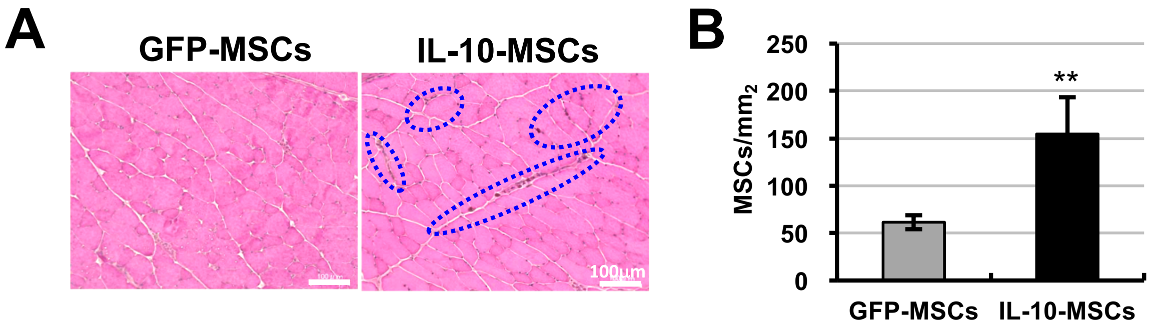

Supplementary Figure. 3

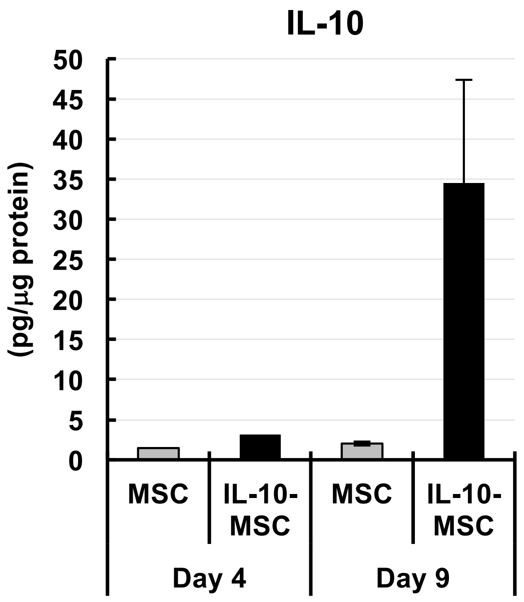

Supplementary Figure 4

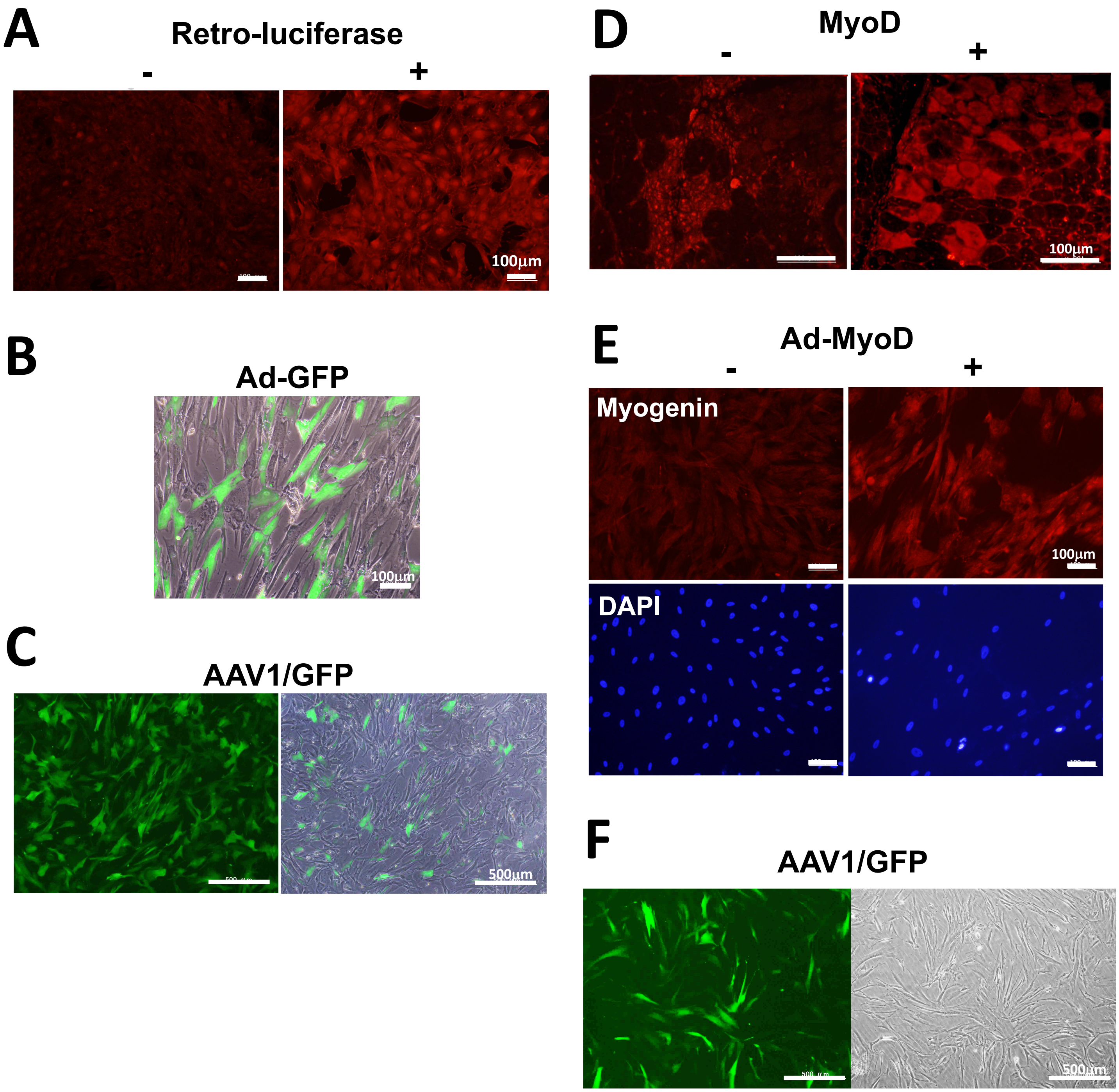

Supplementary Figure 5

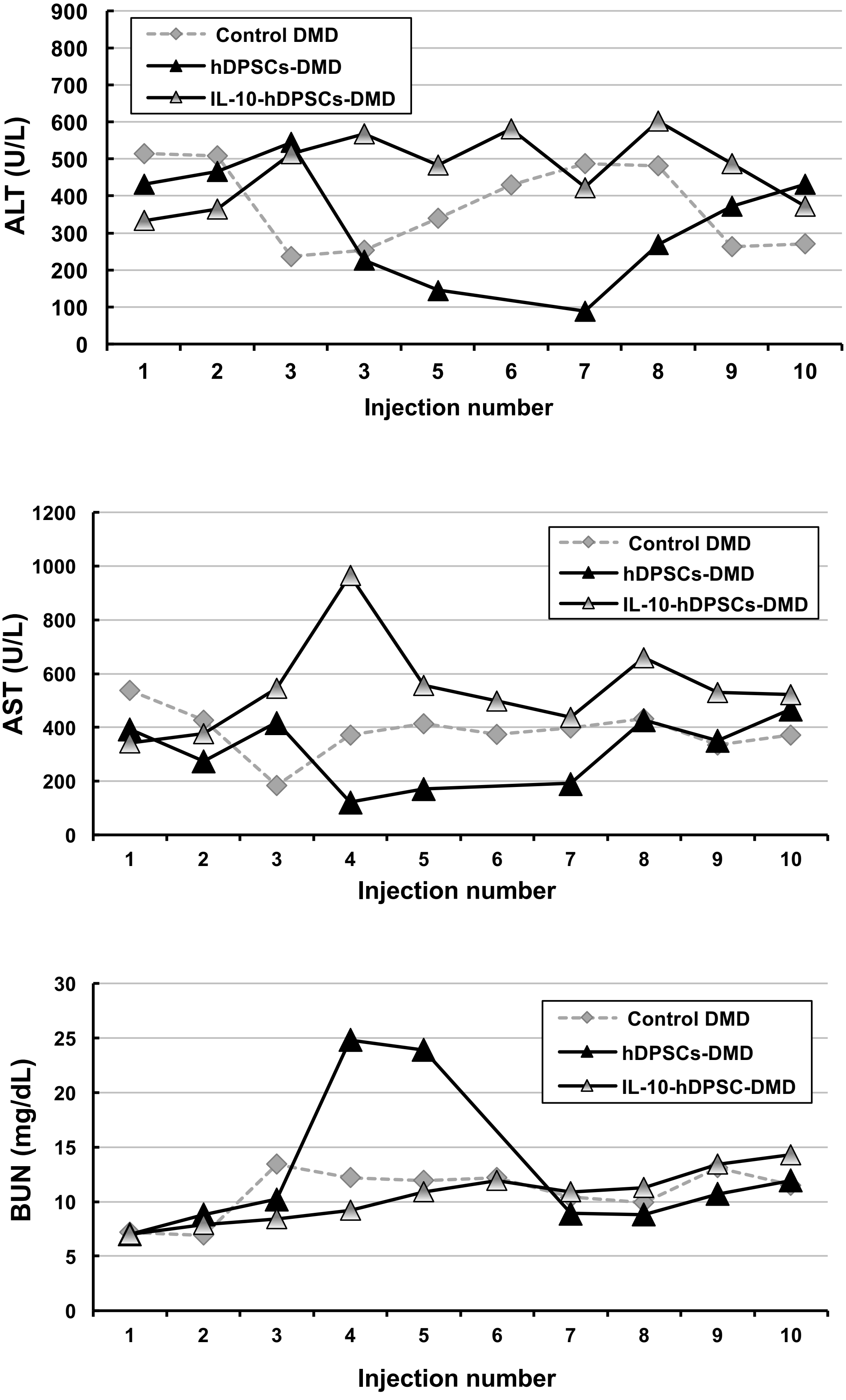

Supplementary Figure 6

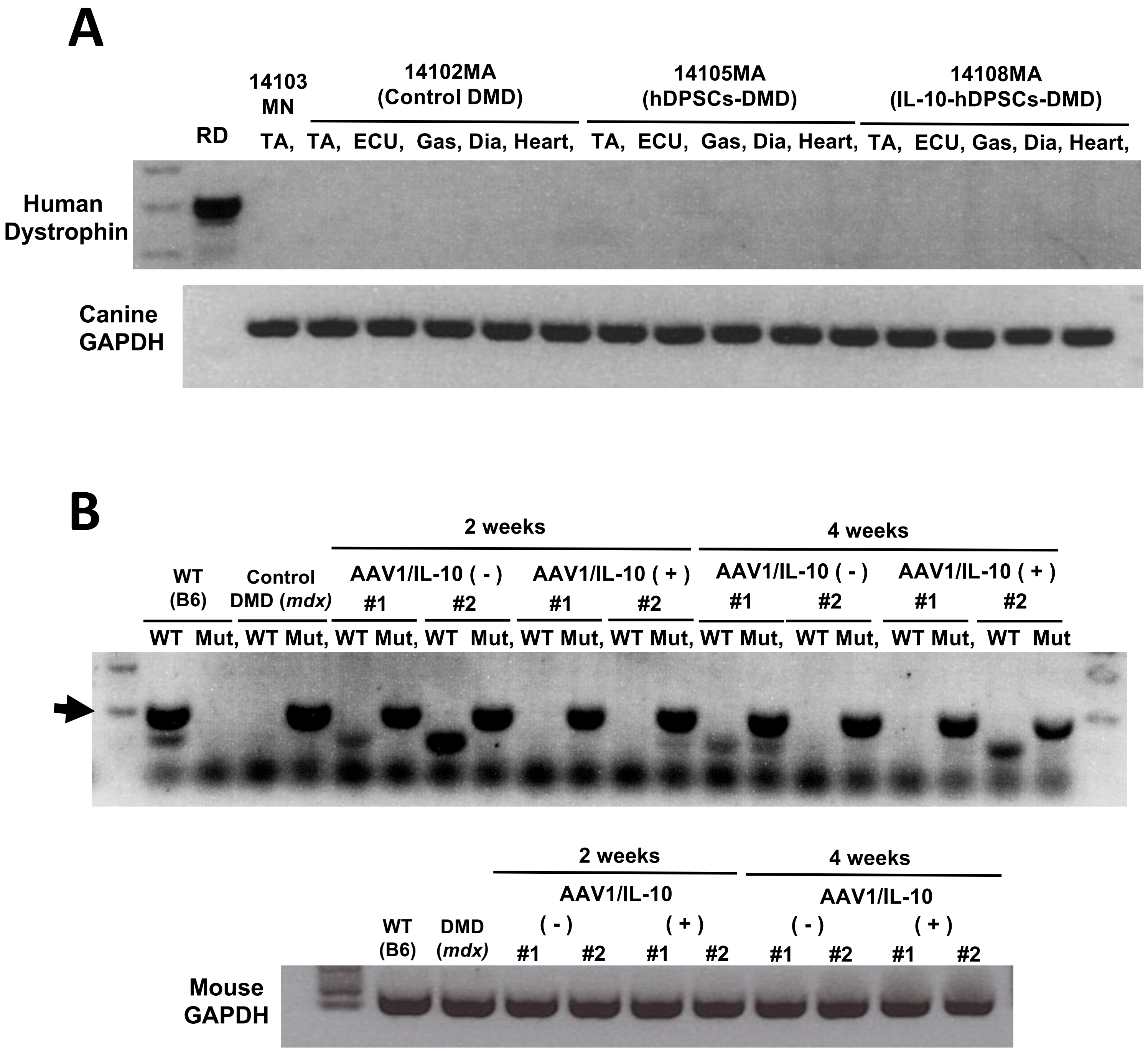

Supplementary Figure 7

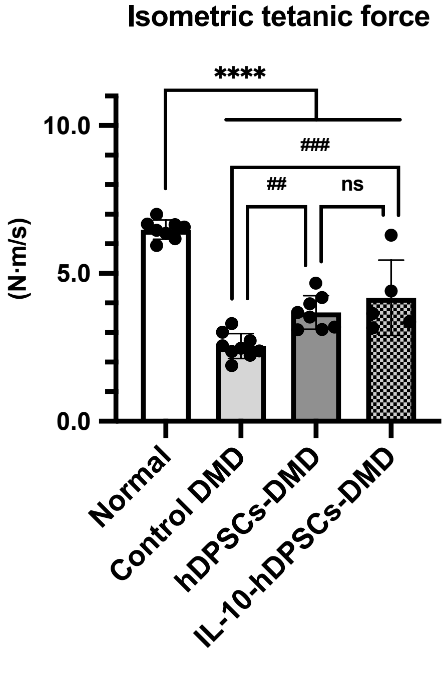

Supplementary Figure 8

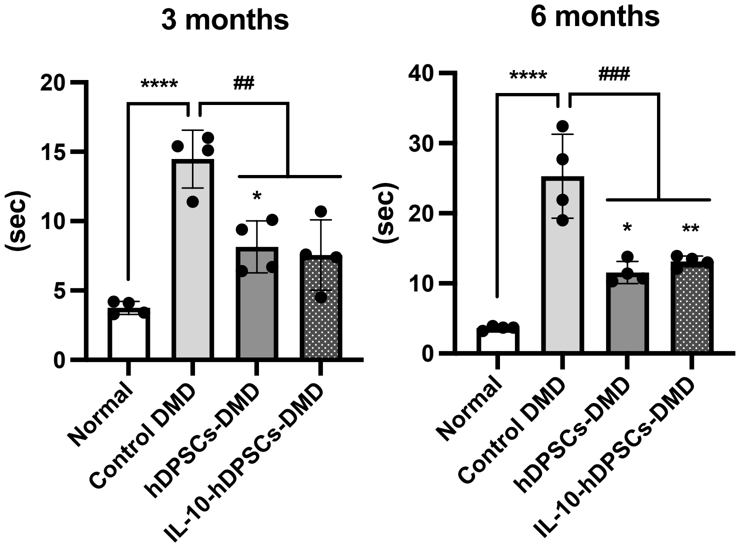

Supplementary Figure 9
